# Optical Platform to Analyze a Model Drug-Loading and Releasing Profile Based on Nanoporous Anodic Alumina Gradient Index Filters

**DOI:** 10.3390/nano11030730

**Published:** 2021-03-14

**Authors:** Pankaj Kapruwan, Laura K. Acosta, Josep Ferré-Borrull, Lluis F. Marsal

**Affiliations:** Departament d’Enginyeria Electrònica, Elèctrica i Automàtica, Universitat Rovira i Virgili, Avinguda Països Catalans 26, 43007 Tarragona, Spain; pankaj.kapruwan@urv.cat (P.K.); laurakaren.acosta@urv.cat (L.K.A.); josep.ferre@urv.cat (J.F.-B.)

**Keywords:** nanoporous anodic alumina, photonic crystals, polyelectrolytes, Rhodamine 6G, optical monitoring

## Abstract

In this work, a methodology that exploits the optical properties of the nanoporous anodic alumina gradient index filters (NAA-GIFs) has been developed and applied to evaluate in real time the release dynamics of a cargo molecule, acting as a model drug, filling the pores. NAA-GIFs with two photonic stopbands (PSBs) were prepared with one of its stop bands in the same absorption wavelength range of the cargo molecule, whereas the second stopband away from this absorption range. Numerical simulation and experiments confirm that the relative height of the high reflectance bands in the reflectance spectra of NAA-GIFs filled with the drug can be related to the relative amount of drug filling the pores. This property has been applied in a flow cell setup to measure in real-time the release dynamics of NAA-GIFs with the inner pore surface modified by layer-by-layer deposition of polyelectrolytes and loaded with the cargo molecule. The methodology developed in this work acts as a tool for the study of drug delivery from porous nanostructures.

## 1. Introduction

Recent years have seen tremendous growth in the design and engineering of nanoporous structures based on modified anodization strategies. Therefore, it is possible now to control light-matter interaction and to fabricate advanced structures to be used in a broad range of applications in the field of biosensing [1,2,3,4], drug delivery [5,6,7], and photocatalysis [8,9]. Nanoporous anodic alumina photonic crystals (NAA-PCs) fall under the regime of optical materials having light modulation capabilities within the spectral regions (from Ultra-Violet to Infra-Red) allowing easy transport of molecules. This light confining capabilities of PCs can be engineered precisely enough to fabricate multidimensional architectures (1D, 2D, and 3D) depending upon the application [10,11,12].

Since the pioneering discovery of nanoporous anodic alumina (NAA) by Masuda and coworkers, NAA has proven itself to be one of the most promising base templates to be used in fabricating varieties of nanoporous architectures [13,14]. NAA can easily be prepared by simple electrochemical anodization of aluminium in an electrolytic solution and consists of closed hexagonal arrays of nanopores from top to bottom. This process is cost-effective, and multiple batches of aluminium can easily be produced at the lab scale without the need of any heavy pieces of equipment. NAA also provides excellent chemical and physical stability, provides stable optical signals, mechanical robustness, and a wide variety of chemistries that make it one of the widely used templates to be researched [15,16]. Several studies have been conducted in the past to engineer different NAA-PCs structures by modified pulse-like anodization strategies such as pseudo stepwise [17], sinusoidal [18,19], Gaussian [20], saw-tooth [21], and stepwise [22]. These strategies have been utilized efficiently in a precise manner to produce several modified nanoporous photonic architectures such as distributed Bragg reflectors (DBRs) [23,24], gradient index filters [2,9], microcavities [22], band-pass filters [25]. Among these structures, GIFs represent a different class of optical structures where the effective refractive index can be modified in-depth in a sinusoidal fashion. The structure consists of sets of parallel pores perpendicular to the sample surface and with a diameter that shows a continuous periodic modulation. This smooth variation in the effective refractive index results in narrow photonic stopbands (PSBs) that serve as a platform for the detection of several molecules and drugs [26,27].

In sustained drug delivery approaches, the burst release of the target molecule has always been a major challenge; therefore, different stimuli are used nowadays to hold the drug molecules and release them only when triggered by any specific stimulus. In this regards, pH-sensitive mechanisms draw huge attention as they are similar to the pH variations inside the human body. The best example of this can be seen in the gastrointestinal tract where the pH remains acidic (pH-2.0) and changes to basic (pH-5–8) in the intestine. Also, the pH of the normal blood is 7.4 as compared to unhealthy or wound-affected areas where it ranges from pH 5.0–7.4 [28]. Henceforth, polyelectrolyte multilayers (PEM) have been often used to coat the surface of nanoporous structures for the sustained delivery of drug molecules [6,29,30].

One of the most challenging problems when PEM are used in NAA nanostructures is the determination of the degree of drug loading into the pores and the rate of release of the drug-loaded into the nanostructure. In this work, the optical properties of NAA-GIFs are exploited to evaluate such magnitudes. To this end, we propose to obtain NAA-GIFs with two high reflection bands, one of them in the wavelength range where a cargo molecule, acting as a model drug, has a maximum optical absorption, while the other in a wavelength range far from such absorption. As a cargo molecule, we use Rhodamine 6G (Rh6G), which is a cationic dye having high fluorescence properties. It has been widely used as a tracer dye for several sensing [31,32,33] and drug delivery applications [34,35]. This study has been planned to demonstrate the effect of the loading and releasing behavior of the Rh6G dye serving as a cargo molecule inside the NAA-GIFs using optical technology.

To this end, the effective medium of NAA-GIFs have been engineered in a sinusoidal fashion to obtain the PSBs in the visible spectrum. Thereafter, the pore widening was performed to widen the pores of NAA-GIFs followed by the drop/dry loading with the cargo molecule and analysis of the change in relative height of reflectance bands with the UV-Visible spectroscopy. Simulations have been performed to demonstrate the real scenario of this filling pattern. Once the changes between both the PSBs were correlated and verified, this method was applied to demonstrate the pH responsiveness aspect of NAA-GIFs in real-time by modifying it with polyelectrolyte multilayers and same cargo molecule where NAA-GIFs were used as reservoirs for the cargo molecules, PSS/PAH coating was employed as a pH-responsive coating. The presence of polyelectrolyte multilayers helps to prevent the early release of the dye molecules hence providing control capabilities to investigate the nature of release from NAA-GIFs.

## 2. Materials and Methods

### 2.1. Materials

Aluminum foils (thickness 0.5 mm, purity 99.999%) were obtained through Goodfellow Ltd. (Cambridge, UK), ethanol absolute (C_2_H_5_OH, 99.0%, ACS reagent), whereas acetone ((CH_3_)_2_CO, ACS Basic) perchloric acid (HClO_4_, 70% ACS), oxalic acid (H_2_C_2_O_4_, C.N. 144-62-7, M_w_ = 90.03 g/mol), (3-aminopropyl trimethoxysilane, C.N. 919-30-2, M_w_ = 221.37 g/mol), hydrochloric acid (HCl, 37%, C.N.7647-01-0), copper chloride (CuCl_2_, C.N.-10125-13-0, >99%), Rhodamine 6G (C_28_H_31_N_2_O_3_Cl, C.N. 989-38-8, M_w_ = 479.01 g/mol), Polystyrene sulfonate (C_8_H_8_O_3_S, C.N.25704-18-1, M_w_ ≈ 70,000), Polyallylamine hydrochloride (CH_2_CH(CH_2_NH_2_.HCl)*_n_*, (C.N.71550-12-4, M_w_ ≈ 50,000), calcium chloride (CaCl_2_, C.N. 10043-52-4, ≥93% anhydrous), phosphate buffer saline (PBS, bioperformance, pH-7.4) were purchased from Sigma Aldrich (Munich, Germany). Double deionized water (DI) (18.6 MΩ, PURELAB Option Q) was used for all the solutions unless otherwise specified.

### 2.2. Fabrication of NAA-GIFs

NAA-GIFs were fabricated by a one-step electrochemical anodization process by applying a sinusoidal anodization profile (Equation (1)) described here [2]. In brief, the aluminum substrates were treated with acetone, ethanol, and water in sequence to remove all organic impurities present on the surface. Pre-treated aluminum foils were electropolished in a mixture of 4:1 *v*/*v* of ethanol: perchloric acid at 20 V for 5 min, alternating the stirring direction every minute. Electropolished aluminum substrates were then anodized in an electrochemical cell containing 0.3 M oxalic acid at 5 °C under controlled stirring.

**Figure 1:**
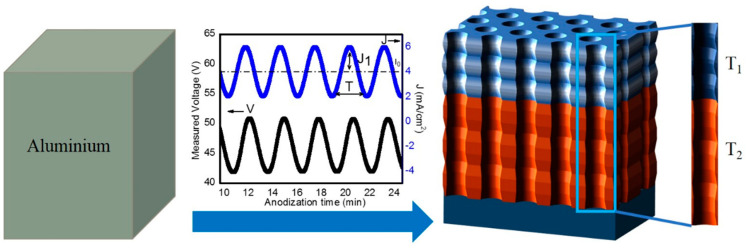
Schematic representation of the electrochemical strategy applied to produce the nanoporous anodic alumina gradient-index filters (NAA-GIFs) through sinusoidal anodization. J_1_ represents current density amplitude, I_0_ represents offset current density, V and J correspond to measured voltage and applied current density respectively, T is the time period of the sinusoidal variation. T_1_ and T_2_ represent the time periods used to obtain the two rugate structures that together form the NAA-GIFs in this study.

All the samples were fabricated by applying a current density anodization profile through Keithley 2400 source meter, with constant monitoring by means of a custom-built LabVIEW^®^ program based on the following Equation [2]:

(1) $$j\left(t\right)=\left\{\begin{array}{c} j_{\mathrm{average}}+j_{\mathrm{amplitude}}\cdot \sin\left(\frac{2\pi }{T_{1}}t\right)\;\mathrm{for}\;0\;<\;t\;<\;N\cdot T_{1}\\ j_{\mathrm{average}}+j_{\mathrm{amplitude}}\cdot \sin\left(\frac{2\pi }{T_{2}}t\right)\;\mathrm{for}\;0\;<\;t\;-\;N\cdot T_{1}<\;N\cdot T_{2} \end{array}\right.$$

where $j\left(t\right)$ is the applied current density at time (*t*), $j_{\mathrm{average}}$ corresponds to the average current density, $j_{\mathrm{amplitude}}$ is the amplitude of the sinusoidal variations of such magnitude. Two different time periods of the sinusoidal time-varying term ($T_{1}$ and $T_{2}$) are used, the first for a time $t_{\mathrm{top}}=N\cdot T_{1}$ while the second for a time $t_{\mathrm{bottom}}=N\cdot T_{2}$, this is for N cycles for each time period (Figure 1). The constant current density and amplitude of the sinusoidal term are the same for the two phases of the anodization. The samples prepared in this study were obtained with $j_{\mathrm{average}}$ = 2.6 mA/cm^2^, $j_{\mathrm{amplitude}}$ = 1.3 mA/cm^2^, $T_{1}$ = 152 s and $T_{2}$ = 210 s. Three different sets of samples were prepared with N = 150, N = 200 and N = 250.

The aluminum layer at the bottom prevents reflectance bands with high contrast. For this reason, it was removed by chemical etching in a mixture of HCl (100 mL in 400 mL H_2_O) and CuCl_2_ (13.6 g added to the HCl/H_2_O mixture). A pore-widening treatment was also performed for all the samples by wet chemical etching in 5 wt% H_3_PO_4_ at 35 °C for 15 min [2]. This treatment widened the average diameter of the pores from the 30 nm of the as-produced samples to 45 nm.

### 2.3. Polyelectrolytes Deposition

**Figure 2:**
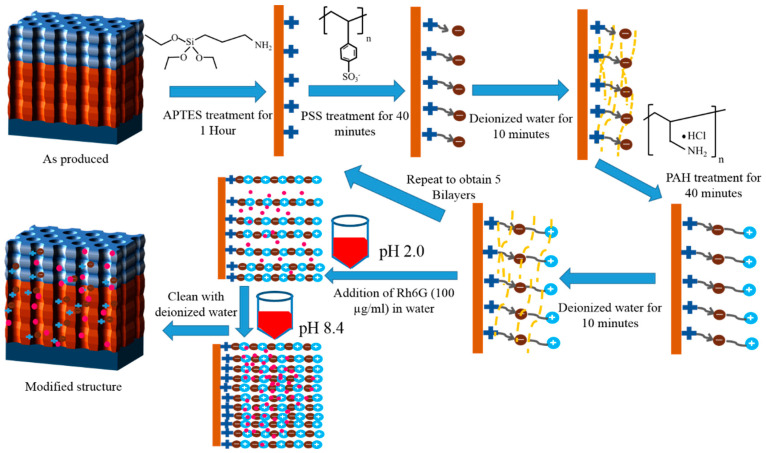
Schematic illustration of polyelectrolyte layer by layer deposition and cargo molecule loading.

To prepare multi-layered NAA-GIFs by layer by a layer deposition method, as-prepared samples were first placed in a mixture of ethanol/water (3:1) having 1% 3-aminopropyl trimethoxysilane (APTES) solution as described here [6]. The samples were kept inside this solution for 1 h under a controlled nitrogen atmosphere and stirring to introduce NH_2_ groups inside the porous structures. To interact with the amine group, samples were first placed in negatively charged polyelectrolyte solution of poly (styrene sulfonate) (PSS, 1 mg/mL in 5 mM CaCl_2_ in deionized water and then, positively charged polyelectrolyte solution of poly (allylamine hydrochloride) (PAH, 1 mg/mL in 5 mM CaCl_2_ in deionized water). The dipping time in each of the polyelectrolytes was 40 min along with the intermediate washing step with deionized water for 10 min before the next dipping (Figure 2). The following steps were repeated 5 times to obtain 5 bilayers.

### 2.4. Rh6G Loading

**Figure 3:**
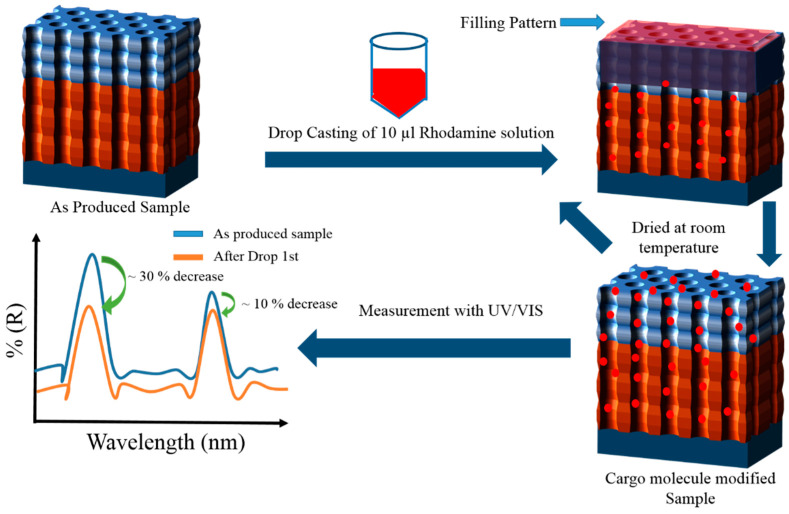
Illustration of drop/dry method performed by drop-casting (10 µL) of Rh6G on the surface of NAA-GIFs followed by the drying procedure in the air and measurement with UV-VIS Spectroscopy. This procedure was repeated for 6 drop/dry cycles for the optical characterization of NAA-GIFs.

For this study, Rh6G was used as a cargo molecule due to its high fluorescence properties and wide use in diagnostic applications. The loading procedure was accomplished by two different methods:

1. In the first method, also denoted as the drop/dry method, the relative height of the photonic stopbands were tested and verified along with the simulations by only using Rh6G dye inside NAA-GIFs. To achieve this, 10 µL drop of the cargo molecule (1 mg/mL) in water was dropped onto the surface of NAA-GIFs and dried at room temperature. In total, 6 drop/dry cycles were performed, followed by measurement with UV-Visible spectroscopy after each cycle (Figure 3). This method does not involve the incorporation of polyelectrolytes or other modifications inside the NAA-GIFs.
2. In a separate set of experiments, keeping in mind the swelling/contraction nature of PSS/PAH polyelectrolytes at acidic and alkaline pH, respectively, NAA-GIFs after the APTES and polyelectrolyte functionalization were first placed in acidic solution of Rh6G dye in water (100 µg/mL) having pH of 2.4 with a mild stirring overnight for the incorporation of dye molecules. Afterward, the pH was changed to 8.4, and samples were stirred for another 3 h, causing molecules to be trapped inside the multilayers. After successful deposition, the samples were thoroughly washed with deionized water to remove unwanted molecules from the surface.

### 2.5. Optical Characterization of NAA-GIFs

**Figure 4:**
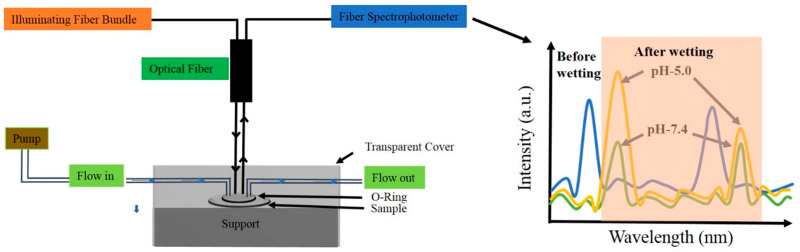
Schematic representation of the flow cell setup used for the real time monitoring of Rh6G dye release under different pH conditions. The intensity vs. wavelength graph on the right-hand side demonstrates the before- and after-wetting effect of the different pH solution on NAA-GIFs and the evolution of the spectra as the pH is changed from 7.4 to 5.0.2.6. Numerical Simulation.

Reflection spectra of NAA-GIFs were measured with a UV-visible spectrophotometer (PerkinElmer Lambda 950) with a Universal Reflectance Attachment (URA) at an incidence angle of 8° with 2 nm resolution in the range of 400–800 nm. Flow cell measurements were conducted using a USB 2000+ fiber spectrometer (Ocean Optics, Orlando, FL, USA). The sample was illuminated by a fiber optic bundle (QR200-7-VIS-BX, Ocean Optics, USA) consisting of six illuminating fibers connected to a tungsten halogen light source (HL-2000-HP-FHSA, Ocean Optics, USA). Light from the illuminating bundle was imaged onto the sample surface by a converging lens in a 4-f configuration. The reflected light was collected with the same lens and directed to a reading fiber in the same bundle connected to the spectrometer. After the dye-loading, the samples were placed inside the flow cell made up of acrylic plastic with a channel size of 2 mm and different pH solutions were flowed at the rate of 140 µL/min in order to detect the pH responsiveness of the system (Figure 4). In the initial 2 h, in order to control the wetting and burst release, PBS with pH 7.4 was flown into the chamber, whereas PBS with pH 5.0 was flown for the next 10 h thus making it a total of 12 h of release. Please note that the mentioned pH values were chosen to register the responsiveness considering the effective pK_a_ values of PAH and PSS, which lie at 8.5 and 1.0, respectively. In addition, the pK_a_ of Rh6G is 6.1. Spectra were recorded from a circular spot having a diameter of 3 mm at 10 s time intervals for the wavelengths between 400 nm and 900 nm employing the Spectrasuite software by Ocean Optics.

### 2.6. Numerical Simulation

For numerical analysis, the calculated reflectance spectra of NAA-GIFs structures filled or covered with the cargo molecule have been obtained with the Transfer Matrix Method (TMM) [36]. TMM is intended for thin-film multilayer structures with definite thicknesses and uniform refractive indices, with the layers divided by interfaces at which a discontinuity of the refractive index exists. This permits the application of continuity boundary conditions and to solve the wave equations at each layer as well as at the incident, substrate, and transmitted media. However, in NAA-GIFs the effective refractive index with the depth of the pore shows a continuous modulation. In order to apply the TMM to the NAA-GIFs structures, it is necessary to discretize such continuous variation of the refractive index into a finite number of uniform layers that approximate the variation of the refractive index.

In the simulations, a stacked NAA-GIFs [2] composed of two structures obtained with two different time periods (T_top_ and T_bottom_) representing one rugate filter stacked on top of another one has been considered. The rugate filters are obtained by applying an anodization current consisting of a constant current density term plus a sinusoidal time-varying term, as specified in Equation (1) above. This applied sinusoidal anodization creates straight pores through the aluminum oxide matrix with a modulation of the pore diameter. In a first approximation, this modulation of the pore diameter can also be considered as sinusoidal, with two different length periods corresponding to the two different time periods, one for the top rugate filter (Λ_top_) and one for the bottom rugate filter (Λ_bottom_). This continuous variation of the pore radius results in a continuous variation of the volume fractions of aluminum oxide and pore contents as a function of the depth, which leads to the continuous variation of the refractive index with the depth into the NAA-GIF structure. In the simulations, each rugate filter consists of 100 periods, being the period length of the top rugate filter Λ_top_ = 164 nm and the period length of the bottom filter Λ_bottom_ = 284 nm. Each period in the rugate filters is discretized into 8 layers. For each of such layers, a value of pore radius is obtained. This pore radius is then used to calculate a value of volume fraction of each of the material constituents at a given depth of the pore: Al_2_O_3_ host matrix, cargo molecule attached to the inner pore surface and medium filling the pores. The average volume fraction for the aluminum host for both rugate structures has been considered as f (Al_2_O_3_) = 0.9, according to the fact that the average anodization current density is the same for both rugate structures. This volume fraction is usually related to the average porosity in the material. 

On the other hand, the sinusoidal variation of the pore radius produces a sinusoidal variation of the aluminum host volume fraction. The amplitude of this sinusoidal variation of the volume fraction has been taken as f (ampl) = 0.025. The amount of cargo molecule into the pores is modeled by assuming a constant volume fraction with the depth as the cargo molecule forms a conformal layer on the pore surface. Different spectra were obtained for cargo molecule volume fractions varying between f (dye) = 0.001 and f (dye) = 0.075. The remaining volume of the pore and the incident medium is considered to be water (the release medium), with a refractive index of 1.33. From the variation in depth of the volume fractions of the constituent materials of the NAA-GIF, the variation in depth of the refractive index is obtained applying the Bruggeman effective medium theory [37] to the mixture of the material constituents (aluminum oxide matrix, release medium, and model drug).

The NAA-GIFs model is completed by including a uniform aluminum oxide layer of about 40 nm thickness at the bottom of the rugate filters to take into account the barrier layer present in the structure as a consequence of its very preparation process. No substrate is considered for the model of the NAA-GIFs structure. The samples are prepared by anodization on aluminum foil substrates. However, the presence of the aluminum substrate provides a high reflectance of the sample in the whole spectral range that reduces the visibility in the spectrum of the high reflectance bands produced by the nanostructuring of the rugate filters. For this reason, the aluminum substrate is removed from the NAA-GIFs structures for flow cell measurement.

The refractive index of aluminum oxide has been taken from the book from Palik [38]. The goal of the numerical study is to determine the impact on the optical properties of NAA-GIFs while it is being infiltrated with the model drug, the refractive index of the drug has not been taken from a particular molecule, but it has been modeled theoretically with the Lorentz Oscillators model [36] for the refractive index (n) and extinction coefficient (k) (Refer to Figure S1 in Supplementary Information). The parameters of the Lorentz Oscillator model have been chosen to obtain a cargo molecule with maximum absorption at the same wavelength range as one of the high reflectance bands of the NAA-GIFs.

### 2.7. Structural Characterization

Structural characteristics, including pore diameter and length analysis were obtained by an environmental scanning electron microscope (ESEM FEI Quanta 600) at an operating voltage of 20 keV. All the images were thoroughly analyzed by ImageJ Software.

## 3. Results and Discussion

### 3.1. Fabrication and Structural Characterization of NAA-GIFs

**Figure 5:**
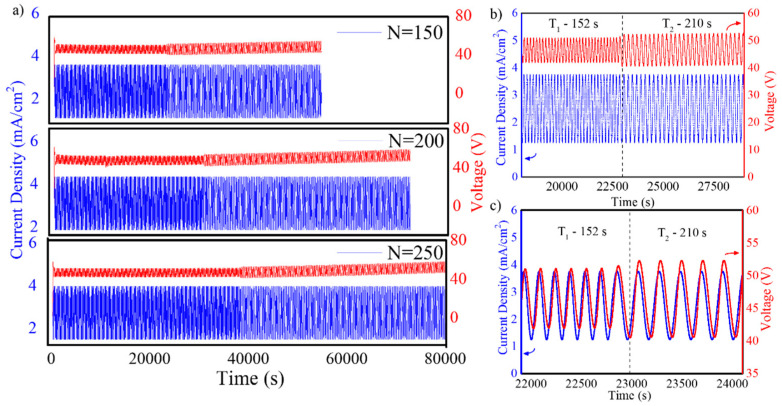
Complete sinusoidal anodization profile applied to obtain NAA-GIFs with parameters j_average_ = 2.6 mA/cm^2^, j_amplitude_ = 1.3 mA/cm^2^; T_1_ = 152 s, T_2_ = 210 s. (**a**) Applied sinusoidal current and resulting measured anodization potential for the different applied number of periods, indicated in the graphs; (**b**,**c**) Detailed view of the data for N = 150 for the time instants between t = 22,000 s–24,000 s, (**c**) Magnified view of the transition region from T_1_ = 152 s to T_2_ = 210 s corresponding to the sample with N = 150.

Sinusoidal anodization profile aims to reshape the pore geometry in NAA-GIFs through continuous modulation of pore diameter in depth. Figure 5a represents the applied anodization current density and measured anodization voltage graphs as a function of time for different samples prepared by applying different numbers of periods, i.e., N = 150, 200, and 250. Figure 5b shows the same magnitude for the sample with N = 150 between the time instants t = 22,000 s–24,000 s. Finally Figure 5c represents the magnified magnitude for the sample with N = 200 between the time instants t = 29,500 s–31,500 s.

Figure 5a depicts the evolution of sinusoidal profile for all the three sample sets fabricated in this study. As there are a large number of periods, the current variation can hardly be distinguished while switching from one period to the next—only a visual change in the curve can be observed. Figure 5b,c show a detail of the applied current and measured voltage where the sinusoidal variation of both magnitudes is demonstrated. In this figure, it can be seen how the sudden change in the period of the applied sinusoidal current produces a discontinuity in the amplitude of the measured voltage. The graph for the measured anodization voltage shows a clear difference between the two time intervals where the periods T_1_ or T_2_ are applied: the value obtained for the measured voltage when a current of period T_1_ is applied is between 41–50 V with an average of 45 V, while when a period T_2_ is applied, the value varies between 40–52 V with an average of 46 V. Furthermore, after the change of period from T_1_ to T_2_, the average value of the measured voltage increases at a rate of 0.02 V/s. This rate increase in the voltage per second was observed for all the different applied number of periods, N. Figure 5c also shows that the measured voltage shows a delay with respect to applied current density for both time periods. This delay is found to be 48 s. 

The above-mentioned results confirmed that by applying a controlled sinusoidal anodization current, the electrochemical system responds with a sinusoidal measured anodization voltage of the same period. As the voltage is mainly applied across the barrier layer at the bottom of the pores and its value is in direct relation with the thickness of the barrier layer, the varying voltage results in a modulation of the pore diameter. The delay of the voltage with respect to the current is explained by the fact that the thickness of the barrier layer increases when current increases and decreases when current decreases, but this response needs some time, as the barrier layer needs this time to adjust its thickness. The change in voltage amplitude can be explained by the fact that for a longer period T, the barrier layer has more time available to adjust its thickness and thus, it can reach a bigger amplitude in thickness, which is translated into a bigger amplitude in the measured voltage [39,40].

**Figure 6:**
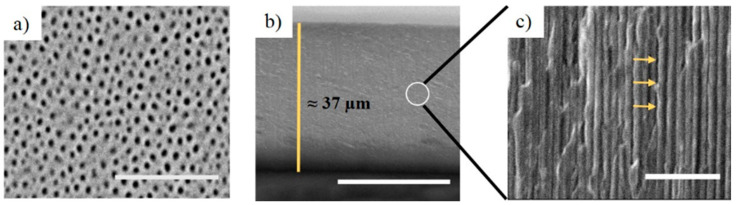
ESEM pictures of as-produced (without any modification) NAA-GIFs for samples with N = 200; (**a**) Top view (Scale bar = 1 μm); (**b**) cross section (Scale bar = 25 μm); (**c**) Magnified view of figure (**b**) showing the modulations in the structure (Scale bar = 1 μm).

Figure 6 represents the structural characteristics obtained through Environmental Scanning Electron Microscopy (ESEM) of NAA-GIFs. Figure 6a shows a top-view ESEM picture of a sample corresponding to N = 200, Figure 6b shows an image of the cross-section parallel to the pores of the same sample, while Figure 6c show a magnified view of the cross-section in b). The cross-section was obtained by breaking the as-produced sample pressing its border with the tip of a tweezer. These images demonstrated the engineered effective medium of NAA-GIFs as a result of voltage change in a sinusoidal fashion. The top view reveals the random distribution of nanopores with the average pore diameter (d_p_) of 45 ± 4 nm and an average inter-pore distance of 116 ± 12 nm (Figure 6a). The cross-sectional ESEM picture of the NAA-GIF reveals a total thickness of approximately 37 µm, corresponding to an anodization time of N = 200 periods with period T_1_ = 152 s and T_2_ = 210 s (Figure 6b). Inset shows the magnified version with the arrows depicting the modulated structure due to sinusoidal pulses (Figure 6c), permitting the desired molecules to move freely within the nanoporous structure.

It can be assumed that during the modification of NAA-GIFs with the LBL deposition, no pore blockage took place. The reason for this is the use of multivalent salt (CaCl_2_) which causes chains to shrink due to electrostatic interaction between the monomer units leading to deposition of the polyelectrolyte layers inside the porous structure [41,42]. To study the effect of modifications on NAA-GIFs inner pore surfaces, a Fourier transform infrared (FT-IR) with attenuated total reflection analysis was also performed in which the spectra was recorded after polyelectrolytes and Rh6G dye modifications. Upon each surface modification, characteristic bands were observed for each deposition confirming the successful modification of NAA-GIFs (Refer to Figure S2 in Supplementary Information).

### 3.2. Optical Characterization and Numerical Modelling of NAA-GIFs

**Figure 7:**
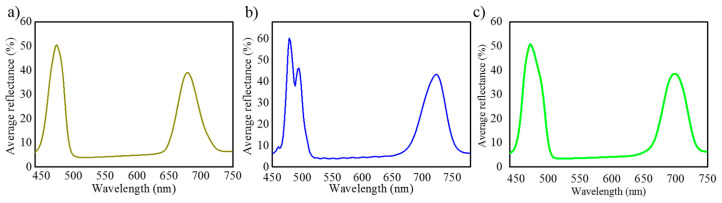
UV-Visible Spectra of NAA-GIFs with varying number of periods, i.e., (**a**) N = 150, (**b**) N = 200, (**c**) N = 250.

The photonic nanostructures prepared in this study were designed to display two stopbands where one of the bands falls within the broad absorption wavelength range of Rh6G dye serving as a “Signal band” while the other stopband is far away from this region, acting as a “Reference band”. To confirm the as-prepared samples show the bands at the desired wavelengths after the pore-widening process, UV-Visible spectra of NAA-GIFs were recorded and measured. Figure 7 shows the obtained spectrum from UV-Vis showing the two expected signal and reference bands for all the time periods in the desired wavelength ranges. In this study for all the samples, the produced reflectance spectra were not recorded, but it can be assumed from the analysis from our group’s previous research that one can expect a blue shift of around 25–30 nm with the 15 min pore-widening process using 5 wt% H_3_PO_4_ at 35 °C [2,20].

Figure 7a shows the reflectance spectrum for the sample fabricated with N = 150, the signal band was positioned at 474 nm. The average reflectance estimated from three different samples is 50.1% and at 678 nm for the reference band with an average reflectance of 38.9%. Similarly, for the sample with N = 200 (Figure 7b), the signal and the reference band were found at 478 and 724 nm with an average reflectance value of 60.1% and 43.2%, respectively. In addition, a side lobe was also observed in this sample, which might occur due to a sharp truncation of the refractive index at the boundaries of the NAA-GIFs [43]. Figure 7c shows the results for samples fabricated with N = 250 where the positions obtained for the signal band were at 472 nm with an average reflectance of 50.6% and 698 nm having an average reflectance of 38.6% for the reference band (Figure 7a–c). With these results, we were able to achieve and confirm one of the bands within the absorption region of Rh6G dye molecule while the other band was obtained far from this region.

**Figure 8:**
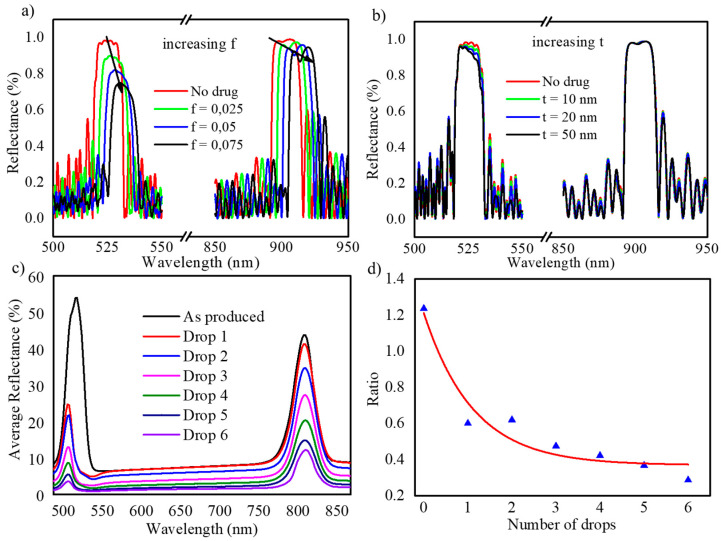
(**a**) Reflectance spectra obtained by numerical simulation considering different volume fractions (f) of a cargo molecule filling the NAA-GIF pores, (**b**) calculated reflectance spectra for different thickness (t) of a cargo molecule layer on the NAA-GIF surface, (**c**) UV/VIS reflectance spectra for NAA-GIFs with N = 200 periods measured after each cycle of the drop/dry experiment; (**d**) points: ratio of the maximum reflectance of the signal band to the maximum reflectance of the reference band, as obtained from the spectra in (**c**) as a function of the number of drop/dry cycles. The line is a non-linear fit to illustrate the trend.

Once the structural parameters were optimized, further analysis was performed for the NAA-GIFs. In this work, we propose to use the two bands of the NAA-GIF as a signal and reference band for the evaluation of the amount of a cargo molecule into the pores. To understand and confirm that this application is possible, a simulation of NAA-GIF structures like the produced ones was carried out. In order to simulate the effect of the cargo molecule filling the pores, NAA-GIFs were simulated with four different volume fractions f (drug) = 0 (pores only filled with water), and f (drug) = 0.025, 0.050 and 0.075. Figure 8a shows the calculated spectra obtained for increasing drug volume fractions. The arrows show that the maximum reflectance of the signal band decreases at a faster rate as compared to the reference band as the amount of molecules within the pores increases. However, the rate of decrease of the signal band corresponding to the drug absorption region is bigger. These two observations can be explained by the increasing amount of drug molecules into the pores, which leads to propose the ratio analysis between the heights of these two maximum reflectance values as a measure for the amount of cargo molecule present inside the pores.

To evaluate whether the formation of a drug layer on the top surface of the NAA-GIFs influences the optical properties of the structure, such formations were also simulated by considering an additional continuous layer of drug with different thicknesses, t = 10 nm, 20 nm and 50 nm. Figure 8b shows the calculated reflectance spectra for the case where the drug molecules form a uniform layer on the top of NAA-GIFs surface. It can be observed that such a thin film on the top of the NAA-GIFs structure has a minor influence on the reflectance, causing only a slight decrease in the maximum reflectance of the signal band. This result can be explained by the fact that even a relatively thick layer of the drug on the top of the NAA-GIFs represents a small amount as compared with the drug that can be filling the pores, even at volume fractions as small as f (drug) = 0.05.

In order to confirm the results of the simulations, drop/dry experiments were conducted using Rh6G as a cargo molecule due to its excellent fluorescence properties. The results showed similar behavior as observed in simulation measurements. Figure 8c,d show the UV-Visible spectra obtained after each drop/dry cycle and the evolution of the ratio of the two reflectance maxima with the number of drop/dry cycles. Figure 8c demonstrates that the addition of the first drop of the dye leads to a drastic change in the effective medium of the NAA-GIFs and the reflectance reduces from about 55% down to approximately 25%. The trend continues to follow as drop/dry cycles are applied. In total, the overall reflectivity was reduced from 54% to 3.5% after the 6th drop addition. Figure 8d shows the ratio between the relative heights of the signal to reference band showing a decreasing trend indicating the successful filling process of the NAA-GIF structure with the dye molecules. This trend indicates that the filling of the pores is not uniform: as the number of drop/dry cycles are repeated, the amount of cargo molecules that can be infiltrated in the pores is exponentially reduced. This can be explained by the fact that the increasing concentration inside the pores makes more difficult the infiltration of further substances.

The above analysis confirms that the simulation results performed are in good agreement with the experimental results and prove that the drop/dry method is useful to infiltrate the NAA-GIFs effectively and to demonstrate a way of analyzing the successful filling of pores. Furthermore, the relative height of the signal band with respect to the reference band can be used as a measure of the relative amount of absorbing species (dye or drug) within the nanopores.

### 3.3. Dye Release Monitoring Using RIfS

**Figure 9:**
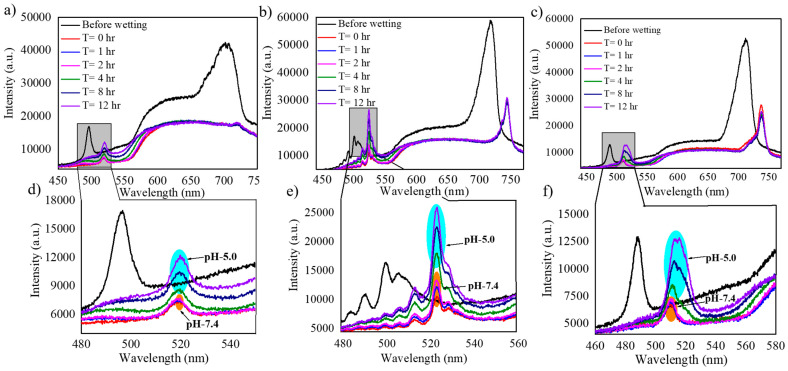
Intensity spectra obtained in the flow cell setup and under two pH conditions (5.0 and 7.4) for NAA-GIFs fabricated with a varying number of periods. (**a**) N = 150, (**b**) N = 200, (**c**) N = 250. (**d**–**f**) represent the magnified view of the signal stopband for the data in (**a**–**c**), respectively.

Dye release was monitored from NAA-GIFs under dynamic flow conditions in a custom-built flow cell. The influence of different pore lengths on the releasing behavior of dye molecules from the pores has been studied. Figure 9a–c shows the intensity spectra measured by the fiber spectrometer (described on the experimental part) at different times of the flow cell release experiments, for samples with N = 150, N = 200 and N = 250 (Refer to Figure S3 in Supplementary Information for the whole release profile). Figure 9d–f represents the wavelength range corresponding to the signal stopband of the same three samples in Figure 9a–c. The first curves in the graphs (black) represent the intensity spectra of the three samples (N = 150, 200, and 250) as they are placed inside the flow cell before wetting. During this state, the intensity spectra show the two high reflectance bands as relative intensity maxima. At a given instant, the PBS solution with pH 7.4 is flowed to fill the cell and to wet the pores. The moment at which the signal stabilizes is taken as the reference time, t = 0. The wetting process induces a redshift on the maximum wavelength of the Signal and the Reference bands, as well as a reduction in the maximum value of such intensity. The spectra taken at instants *t* = 1 h and *t* = 2 h do not show a noticeable variation with respect the one measured at *t* = 0. This corresponds to the period in which the pH 7.4 solution flows into the cell. The successive spectra for t > 2 h show a gradual increase in intensity at the wavelength range of the signal band, while the intensity at the wavelength range of the reference band remains practically unchanged. This time range corresponds with the period in which the solution at pH 5.0 is flowed.

In this study, the release dynamics obtained can be interpreted in three different phases: (a) the wetting; (b) the stabilization; (c) the release phase. The wetting process refers to a process that leads to the wetting of pores with the medium without any release process, which happens because of the use of pH 7.4 solution. In the stabilization phase, the solution with a pH of 7.4 is introduced into the flow cell, and since the solution has a different refractive index than air, it leads to an abrupt change in the effective refractive index of NAA-GIFs resulting in a redshift for both the stopbands followed by a sharp decrease in the maximum signal. This allows the signal to stabilize over time without producing any release. A small increase in signal for the sample with N = 200 is observed during such phase. This can be caused by the cleaning of loosely bound cargo molecules within the structure. 

One of the important aspects of utilizing PSS/PAH multilayers in this study is their pH responsiveness. Herein, when pH is changed closely to the pK_a_ value of the cargo molecule, protonation/deprotonation process of polyelectrolytes takes place, which leads to swelling either by generation or reduction of the charges present in the multilayers [6] [44,45,46]. Figure 9 demonstrates the maximum effect of this under acidic conditions where the final phase takes place, i.e., the release phase. This phase starts by flowing the solution of pH 5.0, due to which the protonation of amine groups present in PAH chain takes place, resulting in an increase in osmotic pressure allowing the cargo molecule molecules to diffuse out through the polyelectrolyte chains. Table 1 enumerates the redshift obtained for the signal band before and after the wetting process during the flow cell procedure for N = 150, 200, and 250.

**Figure 10:**
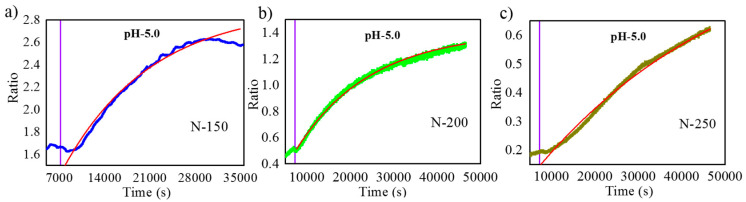
Ratio of the maximum intensity of the signal band to the maximum intensity of the reference band as a function of time, for samples with (**a**) N = 150, (**b**) N = 200, (**c**) N = 250. The line in each graph is the best fit for an exponential function as described in the text.

In order to show the capability of the spectroscopic measurement in the flow cell to evaluate the rate of release, the intensity spectra were registered every 10 s and analyzed. Figure 10 shows the ratio between the maximum of the signal band intensity to the maximum of the reference band of the registered spectra as a function of time. Each of the graphs ((a), (b) and (c)) correspond to samples with N = 150, N = 200 and N = 250, respectively. The three curves show an increasing trend after the introduction of the pH 5.0 solution. For the curve corresponding to N = 150, the increasing trend stops at approximately 7.7 h. Instead, for N = 200, it can be observed that the trend reduces its increase rate with time, but without reaching a stabilization. Finally, for N = 250 the curve also shows a reduction of the increase rate, but with a smaller scale.

All the plots were fitted with an exponential function, i.e.,

(2) $$R\left(t\right)=R_{max}+A\cdot \left(1-\exp\left(-\frac{t-t_{max}}{t_{release}}\right)\right)$$

where *R*(*t*) stands for the ratio as a function of time, $R_{max}$ and $t_{max}$ are taken for each graph as the ratio and time of the maximum measured ratio while the model variables are the Ratio Amplitude (*A*) and the Characteristic Release Time ($t_{release}$). During the analysis, neither points corresponding to the wetting nor the points corresponding to pH 7.4 are included in the fitting. On the other hand, an illustration of the wetting process before and after introducing the solution into the flow cell has been included in the supplementary file (refer to Figure S4 in supplementary information).

The data that can be extracted from this model fitting help in correlating ($t_{release}$) between the different samples. The results of the fitting analysis are given in Table 2.

Since this study mainly focuses on the development of a methodology to evaluate the drug release from different porous nanostructures, the correlation between the characteristic release time and the pore length can be obtained. However, such correlation is not linear: a 33% increase in pore length from N = 150 to N = 200 results in a 27% increase in characteristic release time. Instead, a further 25% increase in length from N = 200 to N = 250 results in a 172% increase in characteristic release time. The correlation between the NAA-GIFs length and the characteristic release time can be explained by the fact that the time needed by the cargo molecule molecules trapped deeper in the pores is longer.

These experiments demonstrate that tailoring the optical properties of NAA in the form of NAA-GIFs permits the evaluation of the release dynamics of the cargo molecule in real time. The analysis performed aims to provide the effect of pH responsiveness of the NAA-GIFs and to establish a method to compare the performance of several different nanostructures used in drug delivery.

## 4. Conclusions

This study successfully demonstrates the ability of NAA-GIFs to be used as an optical platform for analyzing the loading and release of a cargo molecule. To achieve this, NAA-GIFs composed of two photonic stopbands have been obtained, infused with the cargo molecule, and monitored optically for their loading and releasing patterns. Numerical simulation has been applied to predict that the relative height of the signal and the reference PSBs present inside the NAA-GIFs can be correlated with the relative amount of molecules present inside the porous structures. 

Experiments consisting of successive drop-casting the cargo molecule solution followed by the solvent drying were carried out to load the NAA-GIFs with different amounts of drug and confirm the numerical predictions. UV-VIS spectroscopy can analyze the relative height of the high reflectance bands of the PSBs while it is being filled with the cargo molecule. Real-time release measurements were carried out in a flow cell connected to a fiber spectrophotometer that allows measuring the changes in the stopbands during the release process. In this regard, the modification of NAA-GIFs with the polyelectrolytes helps in preventing the burst release of the cargo molecule and thus opens up the possibility to measure the real-time changes in the maximum signal of the NAA-GIFs while different pH solutions are flown within the flow cell, thus permitting a study of the release dynamics from these porous structures. We believe that the methodology developed in this work, exploiting the optical properties of the porous nanostructures can be further applied to the study of drug delivery systems to obtain information about the filling process and real-time data for the releasing process.

## Figures and Tables

**Table 1:** Redshift obtained for the signal band before and after the wetting process for samples with a different number of periods N.

Samples	Wavelength (nm) Before Wetting	Wavelength (nm) After Wetting	Red Shift (nm)
N = 150	496	518	22
N = 200	500	523	23
N = 250	488	509	21

**Table 2:** Characteristic Release Times estimated from the time evolution of the ratio between the signal band intensity and the reference band intensity.

Sample	$t_{release}\;(s)$
N = 150	13,588
N = 200	17,289
N = 250	47,038

## Supplementary Materials

The following are available online at https://www.mdpi.com/2079-4991/11/3/730/s1.
