# Supplementary material for: Optical Platform to Analyze a Model Drug-Loading and Releasing Profile Based on Nanoporous Anodic Alumina Gradient Index Filters
Source: Nanomaterials (Basel). 2021 Mar 14;11(3):730. doi: 10.3390/nano11030730 (PMC7998892; doi:10.3390/nano11030730)
Supplement: Supplementary file 1 [file nanomaterials-11-00730-s001.pdf]

nanomaterials

Supporting information

Optical Platform to Analyze a Model Drug-Loading and Releasing Profile Based on Nanoporous Anodic Alumina Gradient Index Filters

Pankaj Kapruwan, Laura K. Acosta, Josep Ferré-Borrull and Lluis F. Marsal *

Departament d'Enginyeria Electrònica, Elèctrica i Automàtica, Universitat Rovira i Virgili, Avinguda Països Catalans 26, 43007, Tarragona, Spain; pankaj.kapruwan@urv.cat (P.K.); laurakaren.acosta@urv.cat (L.K.A.); josep.ferre@urv.cat (J.F.-B.)

* Correspondence: lluis.marsal@urv.cat

Simulations

The extinction coefficient shows a maximum absorption at 530 nm with a full width at half-maximum of 150 nm, and a maximum absorption coefficient of 0.2. These optical absorption properties are compatible with several drugs or dyes that could be infiltrated and immobilized into the pores. The periods of the rugate filters composing the NAA-GIF have been chosen so that one of the high reflectance bands lies in the range of the high absorption of the model drug while the other high reflection band lies far from this high absorption range.

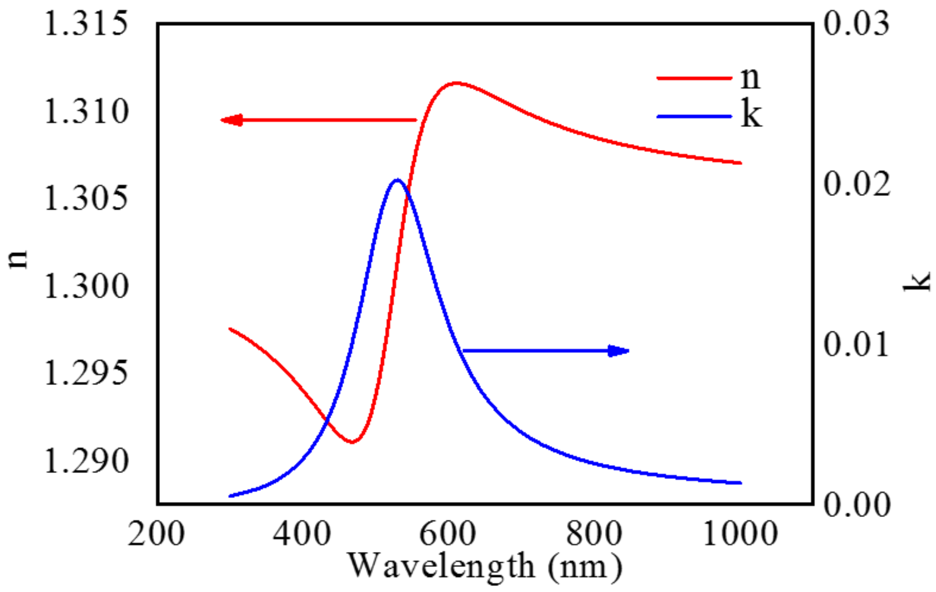

Figure S1. The refractive index (n) and extinction coefficient (k) for the model drug modelled by Lorentz Oscillators model and used in the numerical simulations.

FT-IR Analysis

Figure S2 represents FT-IR spectra of (a) as-produced, (b) after incorporation with 5 Bilayers (PSS/PAH) and (c) after Rh6G dye infiltration inside NAA-GIFs. Curve (a), corresponding to as-produced NAA-GIFs, shows a weak absorption band at 3420 cm^{-1} due to the −OH stretching. Upon modification with multilayers of PSS/PAH, curve (b), bands appear at 1123 and 1170 cm^{-1} representing SO^{3-} stretching vibrations. Another significant band can be observed at 2927 cm^{-1} indicating typical alkyl CH_2 symmetric stretch due to incorporation of PSS along with PAH. In addition, a strong and broad band appears at 3420 cm^{-1} indicating the O-H stretching vibration. Due to the presence of characteristic aromatic ring in the structure, Rh6G shows a broad band between 1400–1530 cm^{-1} and at around 1600 cm^{-1} due to presence of carbonyl group (curve c). Apart from this, another significant C-H stretching band can be observed at around 2970 cm^{-1}. Once the dye was deposited inside the polyelectrolyte layers, the bands representing it are difficult to interpret.

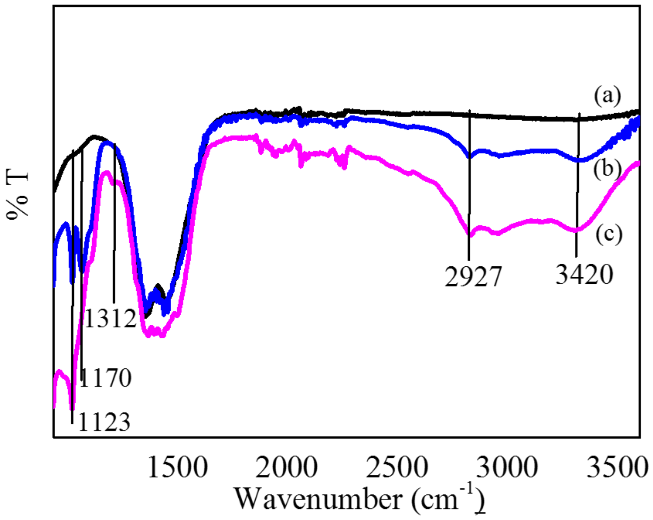

Figure S2. FT-IR analysis of NAA-GIFs after each deposition. (a) as-produced, (b) PSS/PAH coated (5 Bilayers), (c) after Rh6G modification.

Flow cell release

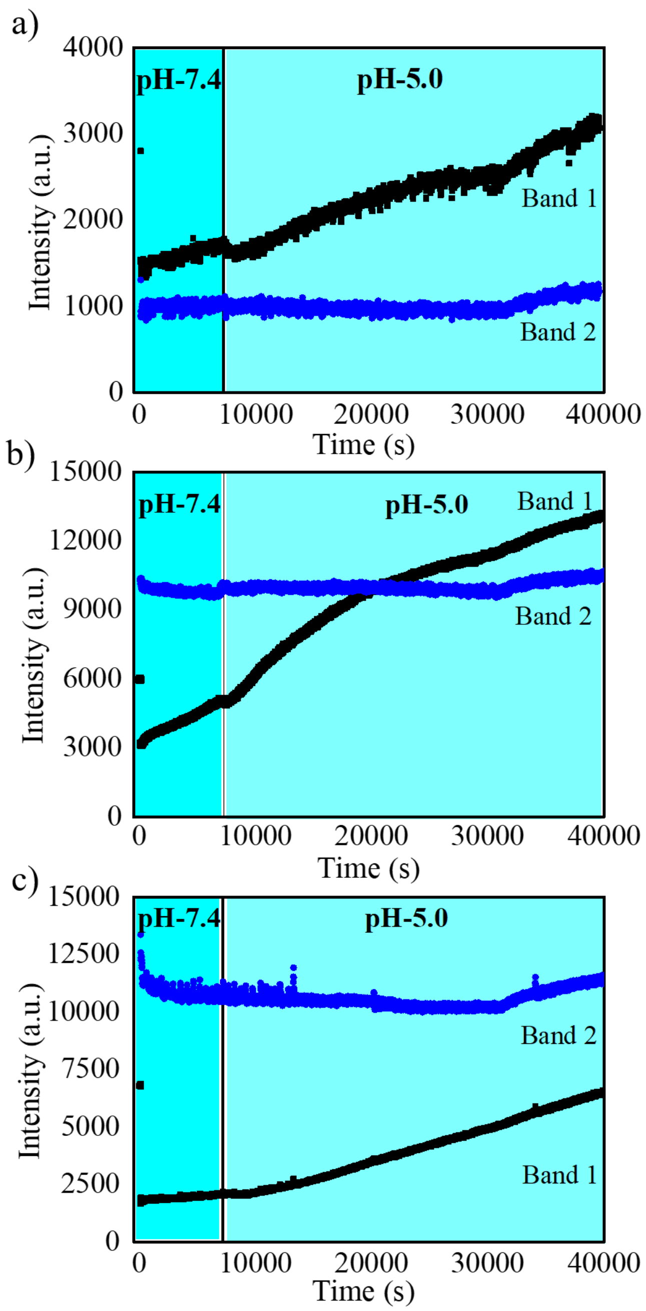

Figure S3. Maximum intensity of the signal band (Band 1) and of the reference band (Band 2) as a function of time. The maximum intensities are obtained in real time from measured intensity spectra of NAA-GIFs in the flow cell and as the model drug is being released. **a**) N=150, **b**) N=200, **c**) N=250.

NAA-GIFs modification with UV-Vis spectroscopy

Treating NAA-GIFs with several modification steps alters the effective refractive index inside the porous structure causing the molecules to absorb the light, hence reducing the overall reflectance. Once the dye is released from the structures, the average reflectance increases. However it does not reach its original value since there is still some amount of dye present inside the NAA-GIFs.

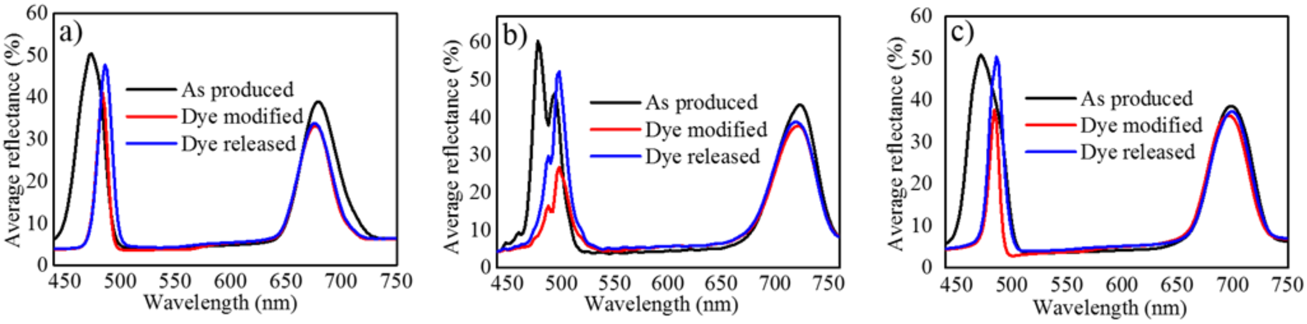

Figure S4. UV-Visible spectrum of modified NAA-GIFs with different number of periods i.e. **a**) N = 150, **b**) N = 200, **c**) N = 250.

Table S1. Maximum average reflectance for the signal band after each modification step.

Time periods	As produced (%)	Dye modified (%)	Dye released (%)
N = 150	50.44	41.02	47.70
N = 200	60.34	26.76	52.21
N = 250	50.82	37.59	50.28
